# Potential Therapeutic Benefit of Combining Gefitinib and Tamoxifen for Treating Advanced Lung Adenocarcinoma

**DOI:** 10.1155/2015/642041

**Published:** 2015-01-26

**Authors:** Chien-Ming Liu, Kuo-Liang Chiu, Tzu-Sheng Chen, Shang-Miao Chang, Shu-Yun Yang, Li-Hsiou Chen, Yung-Lun Ni, Yuh-Pyng Sher, Sung-Liang Yu, Wen-Lung Ma

**Affiliations:** ^1^Sex Hormone Research Center, Graduate Institute of Clinical Medical Science, School of Medicine, China Medical University, 6 Xueshi Rd., North District, Taichung 40403, Taiwan; ^2^Department of Chest Medicine, Taichung Tzuchi Hospital, The Buddhist Tzu Chi Medical Foundation, Chiayi, Taiwan; ^3^Department of Pathology, Taichung Tzuchi Hospital, The Buddhist Tzu Chi Medical Foundation, Chiayi, Taiwan; ^4^Center of Molecular Medicine, China Medical University Hospital, Taichung, Taiwan; ^5^Department of Clinical Laboratory Sciences and Medical Biotechnology, National Taiwan University College of Medicine, Taipei, Taiwan

## Abstract

*Introduction*. Epidermal growth factor receptor (EGFR) mutations are known as oncogene driver mutations and with EGFR mutations exhibit good response to the EGFR tyrosine kinase inhibitor Gefitinib. Some studies have shown that activation of estrogen and estrogen receptor *α* or *β* (ER*α*/*β*) promote adenocarcinoma. We evaluated the relationship between the two receptors and the potential therapeutic benefit with Gefitinib and Tamoxifen. *Methods*. We assessed the association between EGFR mutations as well as ER*α*/*β* expression/location and overall survival in a cohort of 55 patients with LAC from a single hospital. PC9 (EGFR exon 19 deletion mutant; Gefitinib-vulnerable cells) and A549 (EGFR wild type; Gefitinib-resistant cells) cancer cells were used to evaluate the in vitro therapeutic benefits of combining Gefitinib and Tamoxifen. *Results*. We found that the cytosolic but not the nuclear expression of ER*β* was associated with better OS in LAC tumors but not associated with EGFR mutation. The in vitro study showed that combined Gefitinib and Tamoxifen resulted in increased apoptosis and cytosolic expression of ER*β*. In addition, combining both medications resulted in reduced cell growth and increased the cytotoxic effect of Gefitinib. *Conclusion*. Tamoxifen enhanced advanced LAC cytotoxic effect induced by Gefitinib by arresting ER*β* in cytosol.

## 1. Introduction

Lung cancer is the leading cause of cancer-related death worldwide [1, 2], and approximately 80% of cases of lung cancer are non-small cell lung cancer (NSCLC) [1], with lung adenocarcinoma (LAC) being the most prevalent type. However, there are few treatment options for patients with advanced LAC [1, 3]. Driver mutations in NSCLC include EGFR (epidermal growth factor receptor) [4], K-ras (v-Ki-ras2 Kirsten rat sarcoma viral oncogene homolog) [4], ALK (anaplastic lymphoma kinase) [4], ROS1 (c-ros oncogene 1) [4], and Rb (retinoblastoma) [4]. Studies have shown that EGFR mutations are the most “druggable” oncogene driver mutations, with deletion of exon 19 and L858R mutation (exon-21) being the most common EGFR mutations associated with good TKI response in NSCLC patients [5, 6].

EGFR mutation rates vary between Western (10%) and East Asian (50%) populations [1, 2, 4, 7–10]. Therefore, it is not surprising that the results of EGFR-TKI trials differ markedly [5, 6]. For example, studies conducted in East Asia have shown that administration of TKIs results in longer survival for patients with NSCLC whereas in Western countries, studies have shown that administration of TKIs does not appear to have a beneficial effect on overall survival [8, 10, 11]. The reasons for this geographic bias of TKI efficacy are not clear. In addition, EGFR-TKI resistance occurs within 6 to 12 months after treatment [8, 11, 12]. It is generally accepted that induced drug resistance, cellular heterogeneity, and clonal selection of treatment-insensitive cells contribute to disease relapse [11].

Female sex is an independent risk factor for NSCLC in East Asia [4, 13]. Female sex hormones play important roles in disease development and the expression and biologic functions of estrogen receptors (ERs) have been reported to play significant roles in the development of cancer in a number of organs including breast [14], prostate [15], ovary [16], liver [17, 18], and lung [13, 19–23]. However, the correlation between the expression of ERs and clinical outcome remains controversial [13, 19–22, 24–28]. Rades et al. reported that expression of ER*α* in tumor cells is a negative prognostic factor for treatment outcome in both sexes [20]. Also, Omoto et al. reported that ER*β* is expressed without ER*α* in human non-small cell lung cancer [25]. Some in vivo studies have demonstrated that lung cancer cells expressing ER*β* show augmented proliferation upon 17-*β* estradiol treatment [3]. Other studies have revealed that the expression of ER*β* is correlated with favorable prognosis in patients with lung adenocarcinoma whereas lack of ER*β* expression is associated with poor outcome [13, 19]. However, studies have demonstrated that antiestrogen therapy can have antiproliferative effects in patients with NSCLC [2, 29].

Estrogen transactivates its receptors ER*α*/*β* from cytosol to nucleus, where they alter expression of target genes [24, 30, 31]. Estrogen/ER signaling has been reported to both promote and suppress a variety of cancers [32]. Selective estrogen receptor modulators (SERMs) are a category of compounds that modulate the activity and expression of ERs in selected cells such as NSCLC [2, 3].

In this study, we evaluated the potential therapeutic benefit of targeting both EGFR and ERs with Gefitinib and Tamoxifen, a selective ER modulator, in patients with advanced LAC.

## 2. Materials and Methods

### 2.1. Patient Enrollment

Consecutive patients with LAC diagnosed between June 2008 and July 2013 were identified using the Tzu-chi Taichung Hospital cancer registry database. Lung cancer pathology was classified according to World Health Organization pathology classification. Inclusion criteria included patients with advanced LAC disease and adequate tissue specimens. Advanced disease was defined as stage IIIb or stage IV disease according to the 7th edition of the American Joint Committee on Cancer, unresectable Stage IIIa disease, and postoperative recurrence. Access to the tissue samples was approved by the Internal Review Board of the Tzu-chi Taichung Hospital (number REC102-33).

A total of 55 East Asian patients with LAC were enrolled, including 9 patients with stage IIIb, 44 patients with stage IV, and two patients with stage IIIa disease. Of the latter two patients, cancer was deemed unresectable during thoracostomy in one patient and inoperable due to severe congestive heart failure and severe pulmonary edema in the other. One of the stage IIIa patients has received concurrent radiation therapy and chemotherapy. Gefitinib 150 mg was given orally as first line therapy every day in EGFR mutation patients. EGFR wild type adenocarcinoma patients received chemotherapy with intravenous pemetrexed 500 mg/M^2^ plus cisplatin 51 mg/M^2^ every 21 days or vinorelbine 60 mg/M^2^ oral use for the weekly schedule days 1, 8, and 15 every 28 days and cisplatin 51 mg/M^2^ every 28 days. Tumor response was assessed at baseline and every 9 weeks according to RECIST 1.1 criteria. All patients had to be followed up for at least one year. Tumor specimens were collected from all patients and stored according to Tzu Chi Hospital IRB protocols. The clinical features of these patients, including age, sex, smoking history, disease stage, tumor differentiation, TTF1, and estrogen receptors (ERs) *α* and *β* expression status, are listed in Table 1.

### 2.2. Cell Culture, Reagents, and Chemicals

Two lung cancer cell lines, namely, PC9 (exon 19 deletion mutation; TKI-sensitive cell line) and A549 (EGFR wild-type; TKI-resistant cell line), were maintained in Dulbecco's modified Eagle medium (DMEM) (Invitrogen) with 10% fetal calf serum and 1% penicillin/streptomycin (Invitrogen) and incubated in a humidified atmosphere of 5% CO_2_ at 37°C. The antibodies used were ER*α* (HC-20, Santa Cruz Biotechnology), ER*β* (H-150, Santa Cruz Biotechnology), and GAPDH (Santa Cruz Biotechnology). The reagents used were Gefitinib (Gef, Astra Zeneca), Tamoxifen (TAM, Astra Zeneca), and Trypan Blue (Sigma-Aldrich).

### 2.3. Western Blots Analysis

The protein extraction and immunoblot assay were performed as previously described [16]. Briefly, cells were washed with 1x PBS and resolved in RIPA buffer (100 mM Tris, 5 mM EDTA, 5% NP40; pH 8.0) with protease inhibitors (1 mM phenyl-methyl sulphonyl fluoride, 1 *μ*g/mL aprotinin, 1 *μ*g/mL leupeptin). Proteins were resolved by SDS-PAGE and then transferred to PVDF membranes. Blocking of nonspecific binding was accomplished by adding 5% nonfat milk. Primary antibodies were applied and then incubated overnight at 4°C. Secondary antibodies were then added and incubated. Signals were enhanced using an ECL chemiluminescence kit (Millipore, US) and detected by ChemiDoc XRS+ (BioRad).

### 2.4. Cell Growth Analysis: Colony Formation Assessment, Colony Counting, and Gefitinib Cytotoxicity Assay Using WST-1

Colony-forming assays for PC9 and A549 cells were performed as previous study described [33]. Briefly, two sets of 1.5 × 10^5^ cells/dish were seeded on 6 cm plates with DMEM in 10% FBS and incubated for 8 days. In one set of cells, 1000 *μ*L of 4% formaldehyde solution was added to fix cells, which were then allowed to incubate at room temperature for one hour. Crystal violet cell staining was then performed. After one hour, crystal violet was washed from the cell culture dish and cell colonies were photographed. The other set of cells was subjected to colony counting.

Cell viability after exposure to Gefitinib and Tamoxifen treatments was measured using WST-1 reagent according to the manufacturer's instructions. Briefly, 10^3^ cells/well were seeded with media (100 *μ*L) in 96-well dishes in 10% FBS and incubated for 8 days. Then, 10 *μ*L of WST-1 solution was added to each well and cells were allowed to incubate at 37°C in an incubator for an hour. Cell viability was then quantified by colorimetric detection in an ELISA plate reader (BECKMAN COULTER PARADIGM Detection Platform) at an absorbance of 450 nm and 690 nm to generate an OD proportional to the relative abundance of live cells in the given wells.

### 2.5. Immunofluorescence Cell Staining

Two sets of cells were placed in sterile chamber slides overnight at 37°C and then incubated with designed reagents for 18 hours. Cells were then washed with PBS for 5 mins, fixed with ice-cold 99% methanol for 1 min, and then incubated with ER*β* primary antibody overnight at 4°C in a shaker. Cells were then incubated with FITC-conjugated 2nd-antibody for 1 hr at 4°C. After washing, specimens were mounted on coverslip slides in mounting medium containing 1 ng/mL DAPI (Invitrogen) and 50% v/v glycerol in PBS. Fluorescent images were obtained using fluorescence microscopy (Nikon, 80 i, Tokyo, Japan).

### 2.6. Immunohistochemical Staining and Scoring

The immunohistochemistry procedures were performed as previously described with minor modifications [33]. Three-micrometer-thick sections were sliced from paraffin-embedded specimens, deparaffinized in xylene and hydrated in a graded series of ethanol, placed in 0.01 mol/L citrate buffer (pH 6.0), and then autoclaved at 121°C for 10 minutes. Specimens were incubated for 30 mins at room temperature with polyclonal anti-ER*α* antibody and anti-ER*β* antibody diluted 1 : 100 in phosphate-buffered saline. Specimens were then incubated with anti-rabbit horseradish peroxidase-conjugated secondary antibody. Formalin-fixed, paraffin-embedded normal breast tissue was used as the positive control. For the negative control, we used normal colon tissue specimens.

Staining was scored according to the Allred scoring system [19, 34]. Six degrees of proportional score for positive staining were assigned according to the proportion of positive cancer cells (0, none; 1, <1/100; 2, 1/100 to 1/10; 3, 1/10 to 1/3; 4, 1/3 to 2/3; and 5, >2/3). Then four degrees of intensity score were assigned according to the intensity of staining (0, none; 1, weak; 2, intermediate; 3, strong). The proportional scores and intensity scores were then added together. ER expression in tumor cells was categorized as 0, negative; 1 to 5, weak expression; and 6 to 8, strong expression. The slides were independently examined by two of the authors (TS Chen and CM Liu) who were blinded to the clinicopathologic data. When discrepancy was found between sample readings, a consensus was achieved via third pathologist simultaneous examination using double-headed microscope.

### 2.7. EGFR Mutation Analysis

Mutation analysis of the EGFR gene was conducted as described previously [35]. In brief, DNA was extracted from paraffin tissue samples using a DNA extraction kit (Arcturus PicoPure) and the tyrosine kinase domain of EGFR was amplified by polymerase chain reaction. The amplicons were purified and sequenced by an automatic ABI PRISM DNA analyzer with technical support from TR6 pharmacogenomic lab, MOST Taiwan [35]. Two types of EGFR mutations were evaluated with direct sequencing, namely, the deletion in exon 19 and the L858 point mutation in exon 21.

### 2.8. Statistical Analysis

Statistical analyses were performed using PASW statistics version 18 for Windows. Groups were compared with the *χ* ^2^ test. Overall survival was calculated using the Kaplan-Meier method and checked using the log-rank test. A *P* value less than 0.05 was considered to indicate statistical significance. Cox proportional hazards regression model was used to compare the outcomes between different risk factors such as age, sex, smoking habit, stage, tumor differentiation, TTF1, and EGFR mutation status. We calculated hazard ratios (HR) along with 95% confidence intervals (CI) using a significance level of 0.05. A two-sided *P* value less than 0.05 was considered to have statistical significance.

## 3. Results

### 3.1. Cytosolic ER*β* Expression Is Associated with Better Overall Survival

Of the 55 enrolled patients (30 women and 25 men), 20 (37%) were aged ≤60 years. Most were never smokers (*n* = 32, 58%) and 17 were ever smokers. The remaining six patients continued to smoke even after receiving a diagnosis of malignant lung cancer. The majority (*n* = 45, 82%) of patients tested positive for TTF1. Regarding EGFR mutation status, 10 (18%) harbored exon 19 deletions and 14 (25%) had L858 mutations. The majority (*n* = 31, 57%) of patients, however, did not harbor EGFR mutations. Of the 55 patients, 38 (69%) tested positive for strong ER*α* nuclear expression, 27 (49%) had strong ER*α* cytoplasmic expression, 39 (71%) had strong ER*β* nuclear expression, and 21 (38%) had strong ER*β* cytoplasmic expression (Table 1). The initial concordance rate of ER*α* and ER*β* was 86%. Most discrepancies were intensity score not proportion score and consensus was made after simultaneous microscope examination.

The relationship between clinical pathologic factors and EGFR mutation status is shown in Table 2. The only variables that differed significantly between patients with positive EGFR mutation status and those with negative EGFR mutation status were gender and strong ER*α* nuclear expression (*P* < 0.05). There were no significant differences between the two groups (strong expression versus weak expression) in cytosolic expression of ER*α*, nuclear expression of ER*β*, or cytosolic expression of ER*β*. There was no distribution difference in ER*β* cytosolic expression and positive EGFR mutation, even in weak expression group.

**Figure 1:**
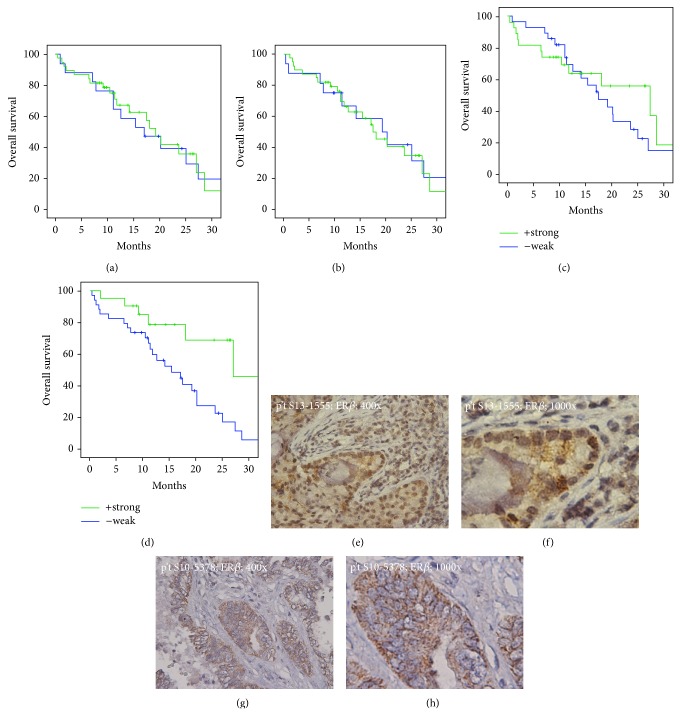
Kaplan-Meier survival curves demonstrated an overall survival benefit of cytosolic ER*β* expression in LAC patients. (a, b) Both nuclear and cytosolic ER*α* expression revealed no significant difference in overall survival in advanced LAC patients. (c, d) Nuclear ER*β* expression exhibited little overall survival benefit in patients. However, cytosolic expression of ER*β* exhibited better overall survival in advanced LAC patients (*P* = 0.018). (e, f) Representative immunohistochemistry staining images of nuclear and cytosolic ER*β* in patient number S13-1555 at lower (400x; (e)) and higher (1000x; (f)) magnification. (g, h) Representative immunohistochemistry staining images of cytosolic ER*β* in patient number S10-5378 at lower (400x; (g)) and higher (1000x; (h)) magnification.

We found that ER*α* localization exhibited little overall survival (OS) benefit (Figures 1(a) and 1(b)). However, as shown in Figure 1(c), nuclear ER*β* expression was not associated with an OS benefit and cytosolic ER*β* expression was associated with good overall survival (log rank test, *P* = 0.005; Figure 1(d)). Of the 21 patients with strong expression of ER*β* in cytosol, 19 also had strong expression of ER*β* in the nucleus. Only the two patients with strong cytosolic expression of ER*β* but without nuclear expression had a longer overall survival than the median survival, although the difference was not significant. However, ER*β* nuclear strong expression (*n* = 39) contributes by 19 ER*β* cytosol strong expressions and 20 weak expressions. The findings reveal the importance of cytosolic expression and location of ER*β*. Figures 1(e) and 1(f) demonstrate strong nuclear and strong cytosolic expression of ER*β* in one of the patients. The images in Figures 1(g) and 1(h) demonstrate strong cytosolic ER*β* expression.

Table 3 summarizes the hazard ratios and significance of the HRs and clinical factors. Cytosolic ER*β* strong expression and female gender were variables of good prognosis and had statistically significant difference in overall survival. Older age was a variable of poor prognosis. EFGR mutation status including exon 19 deletion and L858 mutation had no decreasing or increasing hazard ratios in univariate and multiple analysis.

### 3.2. Gefitinib and Tamoxifen Cotreatment Increases Cytosolic ER*β* Expression in NSCLC Cells

**Figure 2:**
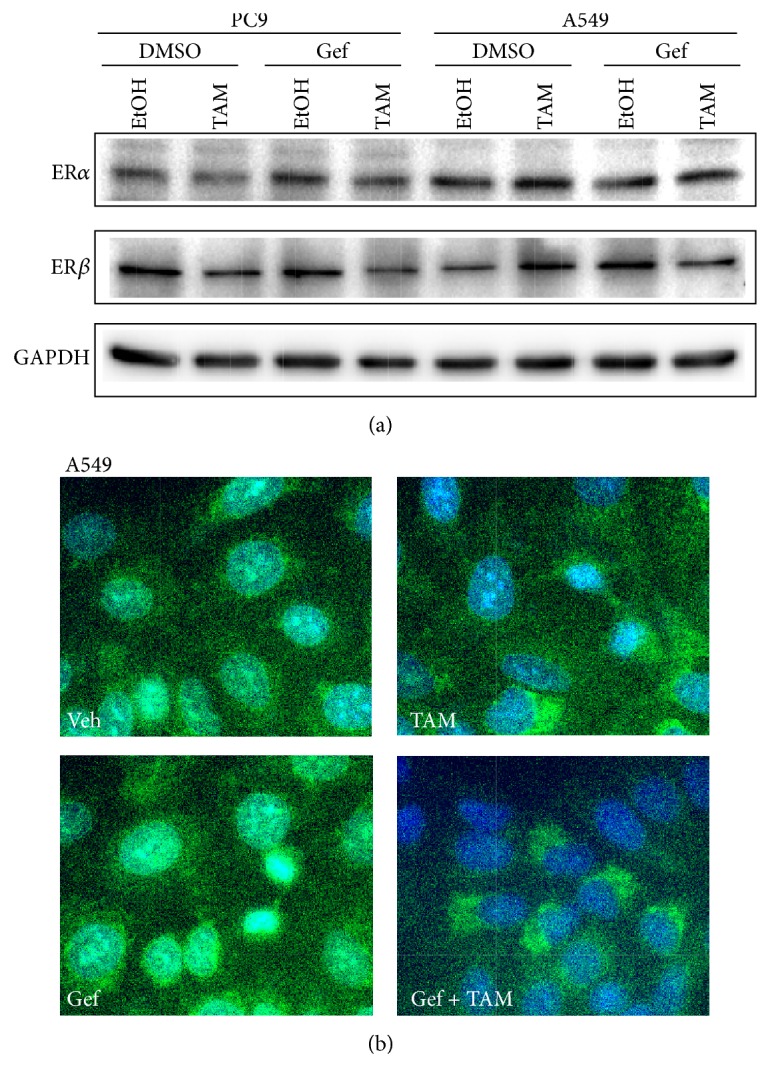
Combination treatment of Gefitinib (Gef) and TAM alters ER*β* cellular localization without changing expression level of ERs. (a) Immunoblot analysis of two LAC cell lines (PC9 and A549) upon Gef and/or TAM treatment. The expression levels of ERs were not altered upon treatments in either cell line. GAPDH served as the loading control in all blots. (b) Gef plus TAM resulted in the relocation of ER*β* from nucleus to cytosol in EGFR wild type A549 cells. The upper left image shows the basal distribution (vehicle treatment; Veh) of ER*β* in nucleus. TAM alone (upper-right image) but not Gef (lower-left image) resulted in a partial reduction in nuclear ER*β* expression. However, combination of Gef and TAM resulted in almost complete retention of ER*β* in cytosol in A549 cells (lower-right images). Representative images of immunoblot assay and immunofluorescence were from at least three reproducible experiments.

We tested whether ablating both EGFR and ER at the same time would result in retention of ER*β* in cytosol. PC9 cells (Gef-sensitive EGFR mutant LAC cells) and A549 cells (Gef-resistant EGFR wild-type LAC cells) were exposed to Gefitinib (Gef, Iressa, the most commonly used TKI in lung adenocarcinoma) and/or Tamoxifen (TAM, the most commonly used SERM). We found that neither ER*α* nor ER*β* expression was affected by Gef, TAM, or Gef + TAM treatments in either cell line (Figure 2(a)). Gef is known to effectively inhibit EGFR mutant PC9 cell growth. In order to test the hypothesis that a combination of Gef and TAM would result in a similar ER*β* localization pattern, we tested whether Gef plus TAM affects ER*β* localization in EGFR wild type A549 cells. As seen in the immunofluorescence images in Figure 2(b), treatment with TAM alone resulted in partially reduced nuclear ER*β* expression (upper-left versus upper-right images) as compared to vehicle. This effect was not seen in cells treated with Gef alone (upper-left versus lower-left images). However, combination of Gef and TAM resulted in almost complete cytosolic accumulation of ER*β* in A549 cells (upper-left versus lower-right images).

Taken together, we found that neither Gef nor TAM alone influenced ER*β* expression but that combination treatment resulted in the relocation of ER*β* from the nucleus to cytosol in EGFR wild type cells. These findings indicate that combined Gef plus TAM treatment might retard the progression of advanced LAC.

### 3.3. Combination of Gefitinib Plus TAM Treatment Reduces Cell Growth and Facilitates Gefitinib Cytotoxic Effect

**Figure 3:**
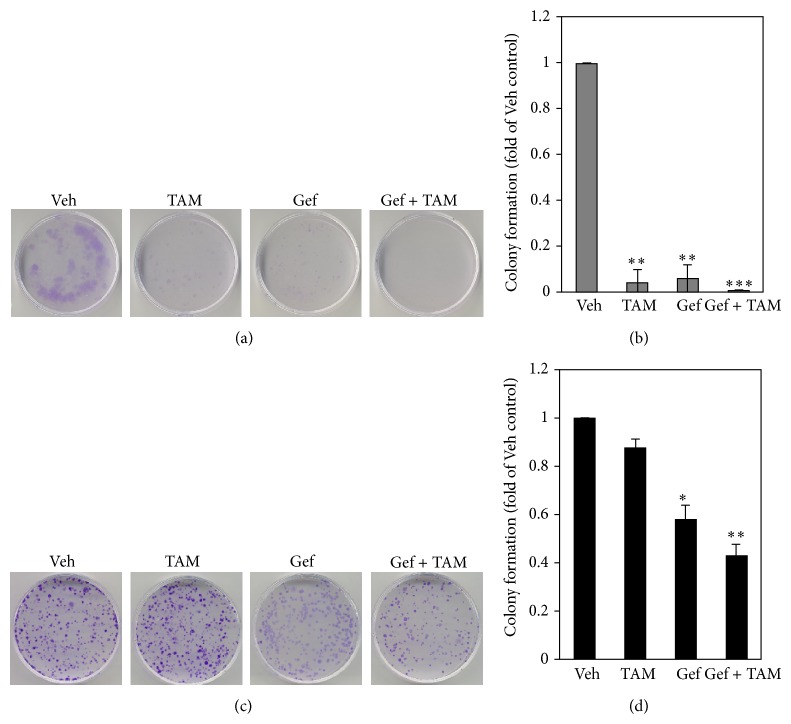
Combination treatment of Gef and TAM resulted in a reduction in LAC cell growth. (a, b) Both Gef and TAM suppressed EGFR mutant PC9 colony forming numbers, while combination treatment further suppressed them. (c, d) Gef and TAM each had a marginal suppression effect on EGFR wild type A549 cell colony forming numbers; however, combination treatment led to significant suppression of colony forming capacity. (a) and (c) are representative sets of images from 3 reproducible independent experiments, where (b) and (d) are the quantitation of results. ^*^Indicating *P* value < 0.05, ^**^indicating *P* value less than 0.01, and ^***^indicating *P* value less than 0.001.

After finding that Gefitinib plus TAM results in cytosolic accumulation of ER*β*, we tested whether combination therapy further suppresses cell growth. As shown in Figures 3(a) and 3(b), Tamoxifen or Gefitinib alone significantly suppressed the colony forming capacity of PC9 cells (1st versus 2nd or 3rd well/bar), and Gef plus TAM further suppressed it (1st versus 4th well/bar). Interestingly, as seen in Figures 3(c) and 3(d), TAM or Gef alone exhibited minor suppression of colony forming numbers of A549 cells (1st versus 2nd or 3rd well/bar), while Gef combined with TAM significantly reduced cell colony formation (1st versus 4th well/bar).

**Figure 4:**
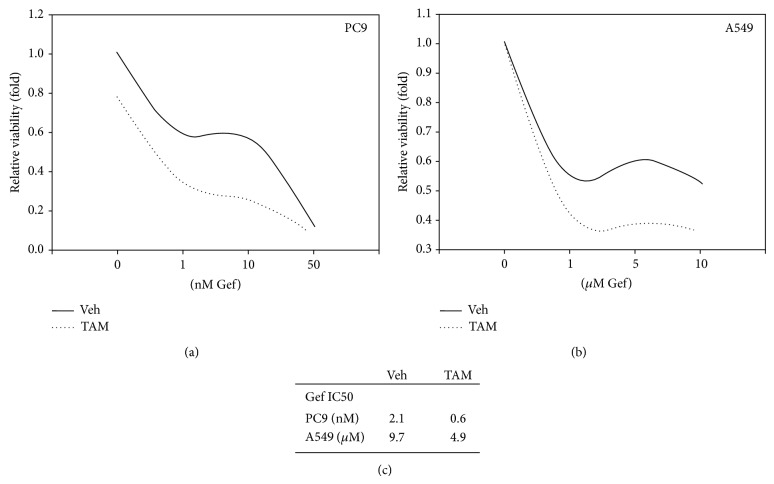
Cotreatment of TAM reduces the IC50 dose of Gef in LAC cells. (a) Cytotoxic effect of Gef on PC9 cells with or without TAM treatment. TAM treatment suppressed cell growth (in 0 nM Gef) and the addition of Gef further suppressed cell growth. (b) Cytotoxic effect of Gef on A549 cells with or without TAM treatment. TAM treatment exerted a limited effect on A549 cells growth; however, the cytotoxic effect of Gef was enhanced by TAM cotreatment. (c) The IC50 of Gef from 2.1 nM to 0.6 nM in the presence of TAM in PC9 cells and from 9.7 *μ*M to 4.9 *μ*M in A549 cells. All results were from at least three independent reproducible experiments.

In order to further characterize the cytotoxic effect of TAM plus Gef, we measured the half maximal inhibitory concentration (IC50) of Gef in the presence or absence of TAM. As shown in Figure 4(a), the cytotoxic effect of Gef on PC9 cells was enhanced when administered with TAM. A similar effect was seen in A549 cells (Figure 4(b)). The IC50 of Gef when administered with TAM decreased from 2.1 nM to 0.6 nM in PC9 cells and from 9.7 *μ*M to 4.9 *μ*M in A549 cells. Taken together, the results indicate that Gefitinib plus Tamoxifen regimen might be an effective therapy for NSCLC.

## 4. Discussion

### 4.1. The Bottleneck of EGFR-TKI Therapy in NSCLC

The study sample size for patients with advanced stage of lung cancer was indeed the major limitation of this study. We initially enrolled 66 patients with confirmed diagnosis of advanced lung adenocarcinoma. Eight patients were excluded due to not advanced stage and receiving lobectomy operation. Another 3 were lacking in adequate biopsy tissue samples. Finally 55 patients were enrolled in our study. Recruiting a sufficient number of the patients is indispensable not only for reducing false-positive results but also for increasing statistical power. Comparing with early lung cancer patients, we are interested in unresectable advanced one. Chemotherapy, target therapy, adjuvant therapy, and determination outcome factors were more important for them. However, our data revealed statistical significance and survival benefit in ER*β* cytosolic strong expressed specimen. It is worthy to conduct a multicenter collaboration study, which will allow increasing sample size of the study cohorts and validating the association in multiple sample sets.

There are very few effective treatments for lung cancer patients. Platinum-based doublet chemotherapy is the mainstay of lung cancer therapy and confers a significant survival benefit compared to supportive treatment. With the development of targeted chemotherapy regimens such as pemetrexed plus bevacizumab, the overall survival can be extended to one year and optimally prolonged to 2 years, particularly in patients with EGFR mutations [1, 11]. However, EGFR-TKI resistance due to T790M mutation and MET (hepatocyte growth factor receptor and encodes tyrosine-kinase activity) gene amplification often occur within 6 months to 12 months [35].

The therapeutic efficacy of EGFR-TKI varies because some EGFR mutations are more common in some populations than in others. For example, approximately half of East Asian patients carry wild type EGFR whereas nearly 70 percent of Caucasians are wild type carriers. Therefore, the antitumor benefit of EGFR-TKI in those patients might be limited. Any therapeutic treatment that improves the effectiveness of TKI intervention, such as compounds that increase EGFR binding affinity in cancer cells, reduce side effects, and reduce the effective EGFR-TKI dose, would help to reach a similar TKI efficacy among different populations [36–38]. ER*β* strong expression is a favorable outcome factor in both resectable and unresectable cases [19, 39]. It got more progression-free survival and overall survival benefit in EGFR mutation patients [19]. Even some report revealed ER*β* strong expression had better treatment response rate and more progression-free survival in EGFR-TKI treatment cases [39]. Its major importance is in unresectable ones. Clinical physicians get significant survival benefit information in EGFR mutation study and ER*β* IHC stain. The clinical practices are popular, easier, and cheaper after long-term breast cancer treatment experience. Besides, it suggests that the expression status of ER*β* can be a candidate surrogate marker for EGFR-TKI treatment of patients with adenocarcinoma of the lung, but not associated with EGFR mutation. Even in cancer cell line study, lack of direct association between EFGR mutations and ER*β* in lung cancer cell line has been reported [40]. The combination of Gef plus TAM even gets treatment response in EGFR wild type cancer cell line study. The data in this study strongly suggest that EGFR-TKI plus SERM additively suppresses EGFR wild type cell growth and results in ER*β* cytosolic retention. In addition, we also revealed, for the first time, that cytosolic ER*β* is associated with better OS in advanced LAC. Our findings also suggest that TAM can mediate the cytotoxic effect of Gef. Moreover, our data imply that patients with cytosolic ER*β* expression are more at risk for EGFR mutation (Table 2) and yet exhibit better response to EGFR-TKI therapy.

### 4.2. Combination Therapy and Cytosolic ER*β* in Lung Cancers

Estrogen signaling plays important roles in many physiological functions and in carcinogenesis, particularly carcinogenesis of mammary epithelial cells [31, 34, 41]. However, only a few studies have investigated the pathophysiological function of cytosolic ERs. For example, Cammarata et al. reported that ER*β* alternative splicing isoforms differentially localized in nuclear/cytosol/lipid raft and were expressed differentially in normal versus transformed lens epithelial cells [42]. Manavathi et al. [43] also reported that ER localization is influenced by HPIP (hematopoietic PBX-interaction protein), a scaffold protein that recruits multiple cellular signaling molecules that influence nuclear receptor function. Furthermore, studies have shown that mitochondrial protein can interact with ER*β* [44] and that TAM can facilitate ER*β* mitochondrial retention, resulting in an increase in cellular oxidative stress in breast tumors [45]. Those findings help explain why Gef plus TAM-related cytosolic ER*β* retention reduces cancer growth. However, those findings do not sufficiently explain how cytosolic ER*β* leads to cytotoxic effects in LAC. Further study is required to explore how ER*β*, a nuclear transcription factor, can function in cytosol.

In summary, combined administration of Gefitinib plus Tamoxifen would retard the progression of advanced LAC by arresting ER*β* in cytosol. Further studies are needed to evaluate whether this combination therapy prolongs time-to-relapse, reduces dose-related complications, and targets the heterogeneity of LAC.

## Figures and Tables

**Table 1:** Clinical characteristics of study population.

Characteristic	Number of patients	%
Age		
>60	35	63%
≦60	20	37%
Sex		
Male	25	45%
Female	30	55%
Smoking history		
Current smoker	6	11%
Ever smoker habit	17	31%
Never	32	58%
Stage		
IIIa	2	4%
IIIb	9	16%
IV	44	80%
Tumor differentiation		
Moderate	36	65%
Poor	19	35%
TTF1		
Positive	45	82%
Negative	10	18%
EGFR		
Exon 19 deletion	10	18%
L858 mutation	14	26%
Unfound	31	56%
ER-*α* nuclear expression		
Strong (≧6)	38	69%
Weak	17	31%
ER-*α* cytosolic expression		
Strong (≧6)	27	49%
Weak	28	51%
ER-*β* nuclear expression		
Strong (≧6)	39	71%
Weak	16	29%
ER-*β* cytosolic expression		
Strong (≧6)	21	38%
Weak	34	62%
Total	**55**	

**Table 2:** Relationship between clinical pathologic characteristics and EGFR mutation.

Characteristic	Number of patients	EGFR mutation^*^		
		Positive	Negative	*P*
Age				
>60	35	15	20	0.550
≦60	20	9	11	
Sex				
Male	25	7	18	0.031
Female	30	17	13	
Smoking history				
Current smoker	6	1	5	0.180
Ever smoker habit	17	6	11	
Never	32	17	15	
Stage				
IIIa	2	1	1	0.760
IIIb	9	2	7	
IV	44	21	23	
Tumor Differentiation				
Moderate	36	17	19	0.327
Poor	19	7	12	
TTF1				
Positive	45	20	25	0.542
Negative	10	4	6	
ER-*α* nucleus expression				
Strong (≧6)	38	20	18	0.041
Weak	17	4	13	
ER-*α* cytosolic expression				
Strong (≧6)	27	14	13	0.175
Weak	28	10	18	
ER-*β* nucleus expression				
Strong (≧6)	39	17	22	0.611
Weak	16	7	9	
ER-*β* cytosolic expression				
Strong (≧6)	21	11	10	0.227
Weak	34	13	21	

^*^EGFR mutation including exon 19 deletion and L858 point mutation in exon 21.

**Table 3:** Hazard ratios of cell expression for mortality risk.

Variables	Univariate model			Multiple model		
	Adjusted HR	95% CI	*P* value	Adjusted HR	95% CI	*P* value
ER*β* cytosolic						<0.001
Weak	1			1		
Strong (≧6)	0.38	0.16–0.87	0.023	0.23	0.07–0.76	0.015
Age	1.07	1.03–1.11	0.001	1.06	1.01–1.11	0.010
Gender						
Female	1			1		
Male	2.17	1.07–4.40	0.031	8.77	2.02–38.00	0.004

Adjusted HR, adjusted hazard ratio; 95% CI, 95% confidence interval.

## References

[1] Siegel R., Desantis C., Virgo K. (2012). Cancer treatment and survivorship statistics, 2012. *CA: Cancer Journal for Clinicians*.

[2] Baik C. S., Eaton K. D. (2012). Estrogen signaling in lung cancer: an opportunity for novel therapy. *Cancers*.

[3] Shim B., Pacheco-Rodriguez G., Kato J., Darling T. N., Vaughan M., Moss J. (2013). Sex-specific lung diseases: effect of oestrogen on cultured cells and in animal models. *European Respiratory Review*.

[4] Seo J.-S., Ju Y. S., Lee W.-C. (2012). The transcriptional landscape and mutational profile of lung adenocarcinoma. *Genome Research*.

[5] Mok T. S., Wu Y.-L., Thongprasert S. (2009). Gefitinib or carboplatin-paclitaxel in pulmonary adenocarcinoma. *The New England Journal of Medicine*.

[6] Fukuoka M., Wu Y.-L., Thongprasert S. (2011). Biomarker analyses and final overall survival results from a phase III, randomized, open-label, first-line study of gefitinib versus carboplatin/paclitaxel in clinically selected patients with advanced non-small-cell lung cancer in Asia (IPASS). *Journal of Clinical Oncology*.

[7] Huang S.-F., Liu H.-P., Li L.-H. (2004). High frequency of epidermal growth factor receptor mutations with complex patterns in non-small cell lung cancers related to gefitinib responsiveness in Taiwan. *Clinical Cancer Research*.

[8] Han S.-W., Kim T.-Y., Hwang P. G. (2005). Predictive and prognostic impact of epidermal growth factor receptor mutation in non-small-cell lung cancer patients treated with gefitinib. *Journal of Clinical Oncology*.

[9] Mendelsohn J., Baselga J. (2003). Status of epidermal growth factor receptor antagonists in the biology and treatment of cancer. *Journal of Clinical Oncology*.

[10] Rosell R., Moran T., Queralt C. (2009). Screening for epidermal growth factor receptor mutations in lung cancer. *The New England Journal of Medicine*.

[11] Chung K.-P., Huang Y.-T., Chang Y.-L. (2012). Clinical significance of thyroid transcription factor-1 in advanced lung adenocarcinoma under epidermal growth factor receptor tyrosine kinase inhibitor treatment. *Chest*.

[12] Yang J.-J., Chen H. J., Yan H. H. (2013). Clinical modes of EGFR tyrosine kinase inhibitor failure and subsequent management in advanced non-small cell lung cancer. *Lung Cancer*.

[13] Wu C.-T., Chang Y.-L., Shih J.-Y., Lee Y.-C. (2005). The significance of estrogen receptor *β* in 301 surgically treated non-small cell lung cancers. *The Journal of Thoracic and Cardiovascular Surgery*.

[14] Schuur E. R., Weigel R. J. (2000). Monoalellic amplification of estrogen receptor-*α* expression breast cancer. *Cancer Research*.

[15] Ye Q., Chung L. W. K., Cinar B., Li S., Zhau H. E. (2000). Identification and characterization of estrogen receptor variants in prostate cancer cell lines. *Journal of Steroid Biochemistry and Molecular Biology*.

[16] Hung Y.-C., Chang W.-C., Chen L.-M. (2014). Non-genomic estrogen/estrogen receptor *α* promotes cellular malignancy of immature ovarian teratoma in vitro. *Journal of Cellular Physiology*.

[17] Yeh S. H., Chen P. J. (2010). Gender disparity of hepatocellular carcinoma: the roles of sex hormones. *Oncology*.

[18] Ma W.-L., Lai H.-C., Yeh S., Cai X., Chang C. (2014). Androgen receptor roles in hepatocellular carcinoma, fatty liver, cirrhosis and hepatitis. *Endocrine Related Cancer*.

[19] Nose N., Sugio K., Oyama T. (2009). Association between estrogen receptor-*β* expression and epidermal growth factor receptor mutation in the postoperative prognosis of Adenocarcinoma of the lung. *Journal of Clinical Oncology*.

[20] Rades D., Setter C., Dahl O., Schild S. E., Noack F. (2012). The prognostic impact of tumor cell expression of estrogen receptor-*α*, progesterone receptor, and androgen receptor in patients irradiated for nonsmall cell lung cancer. *Cancer*.

[21] Kawai H., Ishii A., Washiya K. (2005). Combined overexpression of EGFR and estrogen receptor *α* correlates with a poor outcome in lung cancer. *Anticancer Research*.

[22] Gallo D., de Stefano I., Prisco M. G., Scambia G., Ferrandina G. (2012). Estrogen receptor beta in cancer: an attractive target for therapy. *Current Pharmaceutical Design*.

[23] Navaratnam S., Skliris G., Qing G. (2012). Differential role of estrogen receptor beta in early versus metastatic non-small cell lung cancer. *Hormones and Cancer*.

[24] Siegfried J. M., Hershberger P. A., Stabile L. P. (2009). Estrogen receptor signaling in lung cancer. *Seminars in Oncology*.

[25] Omoto Y., Kobayashi Y., Nishida K. (2001). Expression, function, and clinical implications of the estrogen receptor *β* in human lung cancers. *Biochemical and Biophysical Research Communications*.

[26] Toh C.-K., Ahmad B., Soong R. (2010). Correlation between epidermal growth factor receptor mutations and expression of female hormone receptors in East-Asian Lung adenocarcinomas. *Journal of Thoracic Oncology*.

[27] Rouquette I., Lauwers-Cances V., Allera C. (2012). Characteristics of lung cancer in women: importance of hormonal and growth factors. *Lung Cancer*.

[28] Monica V., Longo M., Felice B., Scagliotti G. V., Papotti M., Novello S. (2012). Role of hormone receptor expression in patients with advanced-stage lung cancer treated with chemotherapy. *Clinical Lung Cancer*.

[29] Stabile L. P., Lyker J. S., Gubish C. T., Zhang W., Grandis J. R., Siegfried J. M. (2005). Combined targeting of the estrogen receptor and the epidermal growth factor receptor in non-small cell lung cancer shows enhanced antiproliferative effects. *Cancer Research*.

[30] Kato S., Endoh H., Masuhiro Y. (1995). Activation of the estrogen receptor through phosphorylation by mitoqen-activated protein kinase. *Science*.

[31] Hershberger P. A., Stabile L. P., Kanterewicz B. (2009). Estrogen receptor beta (ER*β*) subtype-specific ligands increase transcription, p44/p42 mitogen activated protein kinase (MAPK) activation and growth in human non-small cell lung cancer cells. *The Journal of Steroid Biochemistry and Molecular Biology*.

[32] Yao P. -L., Gonzalez F. J., Peters J. M. (2014). Targeting estrogen receptor-beta for the prevention of nonmelanoma skin cancer. *Cancer Prevention Research*.

[33] Chung W.-M., Chang W.-C., Chen L. (2013). MicroRNA-21 promotes the ovarian teratocarcinoma PA1 cell line by sustaining cancer stem/progenitor populations in vitro. *Stem Cell Research & Therapy*.

[34] Hammond M. E., Hayes D. F., Dowsett M. (2010). American Society of Clinical Oncology/College Of American Pathologists guideline recommendations for immunohistochemical testing of estrogen and progesterone receptors in breast cancer. *Journal of Clinical Oncology*.

[35] Su K. Y., Chen H. Y., Li K. C. (2012). Pretreatment epidermal growth factor receptor (EGFR) T790M mutation predicts shorter EGFR tyrosine kinase inhibitor response duration in patients with non-small-cell lung cancer. *Journal of Clinical Oncology*.

[36] Shen H., Liu J., Wang R. (2014). Fulvestrant increases gefitinib sensitivity in non-small cell lung cancer cells by upregulating let-7c expression. *Biomedicine & Pharmacotherapy*.

[37] Xu R., Shen H., Guo R., Sun J., Gao W., Shu Y. (2012). Combine therapy of gefitinib and fulvestrant enhances antitumor effects on NSCLC cell lines with acquired resistance to gefitinib. *Biomedicine & Pharmacotherapy*.

[38] Noto A., de Vitis C., Roscilli G. (2013). Combination therapy with anti-ErbB3 monoclonal antibodies and EGFR TKIs potently inhibits non-small cell lung cancer. *Oncotarget*.

[39] Nose N., Uramoto H., Iwata T., Hanagiri T., Yasumoto K. (2011). Expression of estrogen receptor beta predicts a clinical response and longer progression-free survival after treatment with EGFR-TKI for adenocarcinoma of the lung. *Lung Cancer*.

[40] Onitsuka T., Uramoto H., Tanaka F. (2011). Lack of direct association between EGFR mutations and ER beta expression in lung cancer. *Anticancer Research*.

[41] DeCensi A., Puntoni M., Pruneri G. (2011). Lapatinib activity in premalignant lesions and HER-2-positive cancer of the breast in a randomized, placebo-controlled presurgical trial. *Cancer Prevention Research*.

[42] Cammarata P. R., Flynn J., Gottipati S. (2005). Differential expression and comparative subcellular localization of estrogen receptor beta isoforms in virally transformed and normal cultured human lens epithelial cells. *Experimental Eye Research*.

[43] Manavathi B., Acconcia F., Rayala S. K., Kumar R. (2006). An inherent role of microtubule network in the action of nuclear receptor. *Proceedings of the National Academy of Sciences of the United States of America*.

[44] Zhou Z., Zhou J., Du Y. (2012). Estrogen receptor beta interacts and colocalizes with HADHB in mitochondria. *Biochemical and Biophysical Research Communications*.

[45] Razandi M., Pedram A., Jordan V. C., Fuqua S., Levin E. R. (2013). Tamoxifen regulates cell fate through mitochondrial estrogen receptor beta in breast cancer. *Oncogene*.

